# Polyethylene glycol capped gold nanoparticles ameliorate renal ischemia–reperfusion injury in diabetic mice through AMPK-Nrf2 signaling pathway

**DOI:** 10.1007/s11356-022-21235-5

**Published:** 2022-06-10

**Authors:** Hanan Saleh, Mohamed Salama, Rehab Mohamed Hussein

**Affiliations:** 1 Department of Zoology, Faculty of Science, Cairo University, P.O. Box 12613, Giza, Egypt; 2 Textile Research and and Technology Institute, National Research Centre, El Buhouth street Dokki, P.O. Box 12622, Giza, Egypt

**Keywords:** PEG-AuNPs, Renal ischemia/reperfusion, Diabetes, AMPK, Nrf2, Oxidative stress

## Abstract

The aim of this study is to investigate the protective effect of polyethylene glycol capped gold nanoparticles (PEG-AuNPs) on renal ischemia–reperfusion injury (I/R)–induced acute kidney injury (AKI) in diabetic mice via the activation of adenosine 5′ monophosphate–activated protein kinase—nuclear factor erythroid-2-related factor-2 (AMPK-Nrf2) pathway. Diabetes was induced in male mice (12/group) by streptozotocin (50 mg/kg) for 5 consecutive days. After 4 weeks, the mice have intravenously received doses of PEG-AuNPs (40, 150, and 400 µg/kg body weight) for 3 consecutive days, and then animals were subjected to 30 min ischemia and 48 h reperfusion. Following the treatment with three different doses of PEG-AuNPs, the levels of blood urea nitrogen (BUN) and creatinine were reduced. Obvious reduction in renal tubular atrophy, glomerular damage, mitochondrial damage, and necrotic area were ultra-structurally detected, and renal interstitial inflammation and apoptosis were diminished. Moreover, PEG-AuNPs increased the recovering of damaged renal cells, suppressed significantly levels of malondialdehyde (MDA), downregulated significantly the level of inflammatory cytokines (TNF-α and IL-1β), and upregulated the AMPK-Nrf2 pathway. PEG-AuNPs exhibited a promising alternative therapeutic target for diabetic renal I/R-induced AKI through upregulation of AMPK/PI3K/AKT path which additionally stimulated Nrf2-regulated antioxidant enzymes in a dose-dependent manner.

**Graphical abstract:**

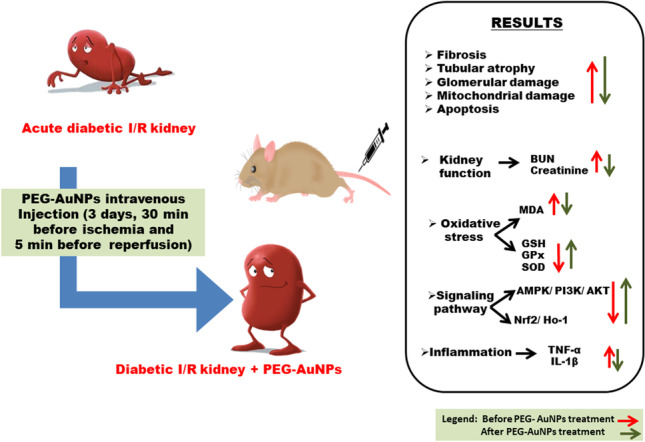

## Introduction

Renal ischemia/reperfusion (I/R) is a momentary preventing of kidney blood supply, followed by re-oxygenation and restoration of blood flow (Đurašević et al. 2019). Renal I/R, whether occurs due to shock or clinical situations including kidney transplantation, partial nephrectomy, vascular surgery, sepsis, and cardiac surgery, is thought to be the most important cause of acute kidney injury (AKI) (Malek and Nematbakhsh 2015). Clinically, the renal ischemia can cause AKI for less than 10 min (Fujii et al. 2019). Despite the magnificent effort made for caring about AKI patients, AKI remains a common and destructive disease correlated with a high death rate (nearly 1.7 million deaths/year) (Zuk and Bonventre 2016; Di Lullo et al. 2017).

Diabetes mellitus (DM) is a metabolic malady featured by hyperglycemia as a result of a variety of cellular and hormonal disorders including deficiency in insulin production or resistance to insulin action, or both (Pickup et al. 2008; Woldu and Lenjisa 2014). Diabetic nephropathy (DN) is the main cause of end-stage renal disease (ESRD) throughout the world (Nordquist et al. 2015). I/R injury in diabetic patients increases notably, when the renal function is impaired, which is indicative of an acute renal failure in severe cases (Kantharidis et al. 2011). A previous study declared that DN is characterized by excessive deposition of extracellular matrix (ECM), thickening of glomerular and tubular basement membranes, and increased amount of mesangial matrix resulting in progressive kidney fibrosis that leads to kidney function decline and irreversible loss of tissue (Liu 2011). During diabetic renal ischemia, the alteration in the tissue architecture occurred due to the detachment of the pericyte, support microvascular stability, from the endothelium, and differentiate into activated myofibroblasts causing vessel rarefaction and unbalanced microvasculature (Schrimpf and Duffield 2011). Moreover, it was confirmed that reactive oxygen species (ROS) increased in mesangial cells (MCs) in high-glucose conditions (Jeong et al. 2012); hyperglycemia can facilitate the production of ROS and amplify its baseline level in various renal cells, including tubular epithelial cells, mesangial cells, and podocytes (Gong et al. 2019). The mitochondria also increases the production of ROS in diabetes, leading to oxidative stress and tissue damage (Pinti et al. 2019). Thus, mitochondrial dysfunction may be a common path of diabetes and I/R injury (Yang et al. 2019). Excessive production of ROS can cause oxidative stress which triggers lipid peroxidation and mitochondrial damage including changes in mitochondrial oxidative phosphorylation, ATP depletion, increase intracellular calcium, and activation of membrane phospholipid proteases (Bonventre 1993; Johnson and Weinberg 1993; Paller 1994). Recent studies have demonstrated that renal I/R is associated with an increment in heart rate and mean arterial pressure (Rahmania et al. 2017); I/R always causes nitric oxide imbalance, neutrophil accumulation, renal tubular epithelial cell damage (Lv et al. 2011, Cho et al. 2013; Yu et al. 2013; Shen et al. 2019), and interstitial fibrosis (Basile et al. 2016); these lead to death by apoptosis and necrosis. Moreover, the production of ROS stimulated the expression of inflammatory factors, including interleukin-6 (IL-6), interleukin-1β (IL-1β), and tumor necrosis factor-alpha (TNF-α), a potent proinflammatory cytokine, which initiates the inflammatory reaction that exacerbates the renal injury (Qi et al. 2016, Liu et al. 2020). The occurring of cellular events during inflammatory responses in renal I/R is always associated with redox balance (Yang et al. 2019).

Nuclear factor erythroid-2-related factor-2 (Nrf2) is a nuclear transcription factor that binds to antioxidant-response element (ARE) and regulates the expression of protective genes in response to oxidative stress, including NAD (P) H, glutathione S-transferase (GSTs), glutathione peroxidase (GPx), superoxide dismutase (SOD), heme oxygenase 1 (HO-1), and γ -glutamylcysteine synthetase (γ -GCS) (Magesh et al. 2012). A recent study displayed that Nrf2 and its downstream antioxidant gene expression were reduced in injured kidney tissue induced by I/R (Li et al. 2018). Previous investigations have demonstrated that the upregulation of Nrf2/HO-1 reduces inflammation and plays a major role in anti-inflammatory function, suggesting that Nrf2 is a therapeutic target in inflammation-associated diseases (Ahmed et al. 2017). In addition, previous studies have proposed that phosphatidylinositol 3-kinase (PI3K)/AKT is a key survival signaling pathway that improves cellular defense and promotes cell survival through modulating Nrf2 as an upstream signaling molecule (Brunet et al. 2001; Nakaso et al. 2003). So, targeting these cellular pathways is attractive to attenuate the renal injury and develop the therapeutic agents.

AMP-activated protein kinase (AMPK) is a metabolic sensor protein that regulates cellular energy metabolism, growth, inflammation, cytokine production, and apoptosis. It is a newly identified regulator of renal hypertrophy in diabetes and its level is inhibited in the diabetic kidneys (Kim and Park 2016). Moreover, a previous study revealed that the AMPK level showed upregulation in the ischemic phase followed by a severe decline in the reperfusion phase during diabetic renal I/R (Declèves et al. 2014a, b). Also, AMPK played protective roles during ischemia by increasing glucose uptake and glucose transporter translocation, decreasing apoptosis through activation of antiapoptotic kinases pathway (PI3K and AKT), and improving postischemic recovery (Hardie et al. 2003). Meanwhile, AMPK downregulated in diabetes-associated kidney disease in both experimental and clinical models, while activation of the AMPK pathway attenuates diabetic nephropathy (Declèves et al. 2014a, b; Li et al. 2017; Hasanvand et al. 2018). In addition, activation of NRF2 by AMPK exerts cytoprotective effects by enhancing anti-inflammatory and/or antioxidative stress responses in diabetes mellitus (Uruno et al. 2013; Yagishita et al. 2014). So, the activation of AMPK pathway diminishes diabetic renal I/R injury through the regulation of energy metabolism, inhibiting oxidative stress, and enhancing Nrf2-mediated antioxidant activity.

Gold nanoparticles (AuNPs) are extensively used in a variety of biomedical purposes, e.g., biosensors, targeted drug delivery (Golchin et al. 2018), immune analysis (Liu et al. 2008), and detection and photo thermolysis of cancer cells and microorganisms (Medley et al. 2008; Bagheri et al. 2018), due to their scattering characteristics and tunable absorption (Daniel and Astruc 2004; Boisselier and Astruc 2009). AuNPs have been used for almost all medical purposes including in vitro diagnostics, prevention, hygiene, and therapy. This enormous utilization of AuNPs is due to their unique physicochemical characteristics, e.g., electronic, thermal, chemical, and biological properties; low cytotoxicity; biocompatibility (Patlolla et al. 2019); and simplicity of their synthesis as well as low cost of preparation in the lab. Moreover, the AuNPs can be easily functionalized by all kinds of biomolecules, such as genes, drugs, and targeting ligands, due to the presence of a negative charge (Fratoddi et al. 2015). Several studies revealed that AuNPs have anti-inflammatory properties as they might inhibit the expression of protein nuclear factor-kappa B (NF-κB) and reduce the level of proinflammatory cytokines TNF-α and IL-1β (Khan and Khan 2018). Also, AuNPs have antioxidant effects and can limit the ROS (BarathManiKanth et al. 2010), and have also antidiabetic potential (Shanmugasundaram et al. 1981). Moreover, AuNPs inhibited high glucose-induced ROS/RNS production, biomolecular damage, antioxidant depletion, and inflammation (Rizwan et al. 2017). Thus, AuNPs may be potential therapeutic agents for ischemic stroke (Zheng et al. 2019) since antioxidants can decrease I/R (Xie et al. 2017). It was shown that uncoated AuNPs are unstable under physiological conditions and can agglomerate which could obstruct small capillaries (Patlolla et al., 2019, Zamora-Justo et al. 2019). Polyethylene glycol–coated AuNPs (PEG-AuNPs) increase their solubility, reduce toxicity and decrease surface charges (zeta potential), and improve the blood stability of AuNPs (Lipka et al. 2010).

Despite substantial advance has been made in the treatment and prevention of I/R injury in diabetic patients, few drugs with potent protective effects and minimal side effects are available. Thus, it is urgent to uncover novel therapeutic agents especially natural substances for the management of I/R injury. The purpose of this study is to declare the effect of PEG-AuNPs in the attenuation of renal I/R injury and the repairing of the affected tissue in diabetic mice in a dose-dependent manner.

## Materials and methods

### Synthesis of AuNPs

A large number of AuNPs can be easily synthesized by the reduction of aqueous chloroauric acid (HAuCl_4_) (Sigma, USA) with sodium citrate (Sigma, USA) (Turkevich et al. 1951). Briefly, all flasks used were cleaned using freshly prepared aqua regia, and then 50 mL of 0.25 mM HAuCl_4_ solution and 34.0 mM trisodium citrate solution was prepared independently. The HAuCl_4_ solution was heated till it reached the boiling point with constant and vigorous stirring. The solution was covered to prevent the evaporation. After the HAuCl_4_ solution reached the boiling point, sodium citrate solution was then added to the boiling HAuCl_4_ solution, and the heating was continued for 15 min to enable complete reaction. The solution was then allowed to cool to room temperature with continuous stirring yielding citrate-capped AuNP. The synthesis was complete when the color of the suspension no longer changed. In this synthesis, citrate ions act as reducing agent and stabilizer (Kong et al. 2017); furthermore, the negative charge of citrate ions repels AuNPs from each other. The size of gold nanoparticles formed depends on the sodium citrate concentration, salt of gold, temperature, and boiling duration (Hussein and Saleh 2018).

### PEG‑functionalized AuNPs

The polyethylene glycol–coated gold nanoparticles were prepared as described previously (Stiufiuc et al. 2013). Briefly, 340 µL of dicarboxylic PEG600 (Sigma-Aldrich, St Louis, MO) and 0.75 mL of NaOH 1% were added to 50 mL of water and stirred at 50 °C. About 20 mg of HAuCl_4_ dissolved in 5 mL dist. Water was added rapidly with stirring for 1–2 h to allow PEG molecules to modify the surface of AuNPs covalently. The resulting solution was then slowly heated up to 70 °C, during that the transparent color of the solution turned into purple-red color, confirming the formation of PEG-coated gold nanoparticles. The resulting PEG-coated gold nanoparticles were purified by centrifugation to remove the excess of non-conjugated PEG. Centrifugation was carried out at 12,000 × g for 25 min three times and washed twice with distilled water. The PEG-coated gold nanoparticle solution was stored at 4 °C to prevent aggregation.

### Characterization of PEG‑AuNPs

A transmission electron microscope (JEM-100CXII, JEOL Ltd, Tokyo, Japan) was used to recognize the size, the morphology, the tendencies of aggregation, and the cellular distributions of AuNPs and PEG-AuNPs. Dynamic light scattering (DLS) was assumed to measure the hydrodynamic diameters and the surface zeta potential of AuNPs using a Nano Zetasizer particle analyzer (Malvern Instrument ZS-Nano, Malvern, UK) at a temperature of 25 °C. Inductively coupled plasma mass spectrometry (ICP-MS, XSENIES, USA) was utilized to determine the concentration of gold nanoparticle solution (µg/mL). An ultraviolet spectrophotometer (UV2550, Shimadzu, Japan) was applied to determine the absorption peaks of coated and uncoated AuNP solutions.

### Experimental animals

A total of 60 male BALB/c mice (10–12 weeks old, weighing 25–28 g) were purchased from the National Research Center animal house, Egypt. They were randomly allocated to polycarbonate cages (12 mice/cage) and adapted for 7 days at 23 ± 3 °C and humidity 51 ± 0.5% with a 12/12 h light/dark cycle and access ad libitum to fresh tap water and a normal rodent diet. The experimental protocol was approved by the Institutional Animal Care and Use Committee (IACUC), Faculty of Science, Cairo University (Egypt) (CUFS/I/F/PHY/66/18). All the experimental procedures were done following the international guidelines Guide for Care and Use of Laboratory Animals.

### Induction of diabetic mouse model

Diabetes type 1 was induced in male mice by intraperitoneal injection of streptozotocin (STZ, Sigma, St Louis, MO, USA) at a dose of 50 mg/kg freshly dissolved in 100 mM citrate buffer (PH 4.5) for 5 consecutive days. Control animals were injected with the same volume of vehicle (citrate buffer). Before the injection, mice had been deprived of food overnight but had free access to water. After 4 weeks, blood glucose levels were measured using an Accu-Chek glucose meter (Roche, USA) by tail vein. Mice with blood glucose levels of > 11.1 mmol/L were used for the current study. Within these 4 weeks, weight changes and blood glucose measurements were recorded.

### Experimental design

The diabetic animals were randomly divided into five groups (12 mice/group) as follows: Diabetic sham (DS) group: the diabetic mice underwent the only laparotomy (abdominal exploration), where the kidneys were manipulated without occlusion. Diabetic renal ischemia–reperfusion (DI/R) group: the diabetic mice subjected to 30 min of renal ischemia followed by 48 h of reperfusion. DI/R + PEG-AuNPs 40 µg/kg group: the diabetic mice received PEG-AuNPs 40 µg/kg body weight 30 min earlier to ischemia and 5 min prior to reperfusion. DI/R + PEG-AuNPs 150 µg/kg group: the diabetic mice received PEG-AuNPs 150 µg/kg body weight, 30 min before ischemia and 5 min before reperfusion. DI/R + PEG-AuNPs 400 µg/kg group: mice received PEG-AuNPs 400 µg/kg body weight, 30 min before ischemia and 5 min prior to reperfusion. The PEG-AuNP-treated animals received dose-dependent PEG-AuNPs intravenously (i.v.) for 3 consecutive days then exposed to 30 min renal ischemia and 48 h reperfusion. The DS and DI/R groups received the same amount with saline for 3 days before the induction of I/R and a single dose of warmed saline was injected i.v. 30 min before ischemia and 5 min prior to reperfusion. The doses of PEG-AuNPs were chosen according to the previous literature and the toxicity test of PEG-AuNPs (Zhang et al. 2010, 2011). In addition, to ensure the harmless effect of PEG-AuNPs, the three different doses (40, 150, and 400 µg/kg) were injected into normal control mice (6 mice/group) and assess the renal function, BUN and creatinine and the gene expression and protein level of Nrf2.

### Renal ischemia/reperfusion (I/R) model

Animals whose hyperglycemia had been successfully induced were randomly assigned to renal I/R injury. Mice will lightly anesthetize by injection of (50 mg/kg) ketamine; with (10 mg/kg) xylazine, animals should display regular respiration. During the operation, the animals were placed on a controlled heating pad keeping the body temperature at 37.5 °C. Midline laparotomy incisions were made along the bilateral vascular pedicles and clamped with a microvascular clip. A visible sign of ischemia onset was a change in the kidney color from pale pink to deep purple. After 30 min of occlusion, the ligation was loosened and then subjected to 48-h reperfusion. In the diabetic sham group, renal pedicles were exposed without any intervention after laparotomy. Animals will resuscitate with 1 mL subcutaneous 0.9% warm saline after the 48-h reperfusion period. Animal’s stress and pain were reduced during the ischemia and reperfusion by intraperitoneal injection of buprenorphine (0.1 mg/kg) (Ban et al. 2013).

### Sample processing

After the experimental period, animals were humanely sacrificed by exsanguination; at the end of the reperfusion period, blood was collected through cardiac puncture and centrifuged at 1008 × g for 10 min. The serum was stored at − 80 °C for the analysis of kidney function. A part from left kidney tissue from all groups was snap-frozen in liquid nitrogen and preserved below − 80 °C for the enzyme-linked immunosorbent assay (ELISA) and antioxidant enzymes. Another part from the left kidney was fixed in 10% formalin for histochemical investigation and stained with routine hematoxylin and eosin (H&E) and Masson trichrome; finally, a part was preserved in 1 mL RNA later and was kept at − 80 °C for assessment of gene expression.

### Histopathological analysis and Masson trichrome staining

After kidney tissue fixation and embedding in paraffin, sections of 4 µm thickness were stained with hematoxylin and eosin (H&E) (Meyer 1903). The deposition of collagen was detected by Masson trichrome stain. The severity of renal injury and collagen deposition was analyzed by two independent histologists blinded to the experimental groups. Sections were examined for histopathological evaluation under the light microscope (Leica DM 2500; Leica, Wetzlar, Germany) with a digital image camera (Leica DFC4200; Leica Germany) (Olympus CX41, Japan). In ischemic AKI, the most severely injured site is the proximal tubules located at the outer stripe of the outer medulla. We routinely conduct H&E staining to grade tubular damage (0, no damage; 1, 0–25% damaged tubules; 2, 25–50% damaged tubules; 3, 50–75% damaged tubules; 4, > 75% damaged tubules) (Brooks et al. 2009; Jiang et al. 2012). Subsequently, the total kidney injury scores were calculated by counting the individual scores for each group.

### Kidney function assessment

The levels of renal function markers including blood urea nitrogen (BUN) and creatinine were determined using the appropriate commercial kits (Bio-Diagnostic, Dokki, Giza, Egypt).

### Kidney semithin and ultrathin section examination

Small pieces of kidney were extracted from each specimen and fixed immediately in 5% cold buffered glutaraldehyde for 24–48 h, cleaned in cacodylate buffer (pH 7.2) 3–4 times for 20 min, postfixed in 1% osmium tetroxide for 2 h, rewashed in the same buffer 4 times, and then dehydrated in alcohol. The specimens were fixed in Epon–Araldite mixture. From the embedded blocks, the semithin sections with thickness (0.5–1 µm) were cut by LKB ultramicrotome (Bromma, Sweden) stained with toluidine blue and photographed by sc30 Olympus camera (Münster, Germany). The ultrathin Sect. (500–700 A◦ thickness) was cut using Leica AG ultramicrotome (Wetzlar, Germany) and stained with uranyl acetate and lead citrate. The stained sections were then examined using the JEM-100CXII operating at 80 kV and photographed by CCD digital camera Model XR-41 (CA, USA).

### Antioxidant system

A part of kidney tissue was homogenized (10% w/v) in ice-cold 0.1 M Tris–HCl buffer (pH 7.4). The homogenate was centrifuged at 1008 × g for 15 min at 4 °C and the resulting supernatant was used for the biochemical analysis. The kidney tissue content of malondialdehyde (MDA) (Ohkawa et al. 1979), reduced glutathione (GSH) (Beutler 1963), and the activities of glutathione peroxidase (GPx) (Paglia 1967) and superoxide dismutase (SOD) (Nishikimi et al. 1972) were measured using Bio-diagnostic assay kits (Giza, Egypt).

### Quantitative real time (qRT-PCR)

Total renal RNA was isolated from the kidney cells of normal, diabetic, and treated mice using Trizol reagent (Invitrogen) and purified by DNase I on RNeasy Mini Elute colonies (Qiagen, RNeasy Mini Kit) and stored at − 80 °C. RNA was reversed transcribed into cDNA by reverse transcriptase Superscript II (Invitrogen). Then cDNA was subjected to an appropriate primer and SYBR Green/ROX PCR mix (Thermo Fisher Scientific, USA). The reaction product was amplified using the Step One Plus system (Applied Biosystem, CA, USA) according to the kit’s manual instructions. The qRT-PCR cycle profile was performed at 95 °C for 10 min, followed by 40 cycles of 15 s at 95 °C with a denaturation temperature, 30 s at annealing temperatures of 60 °C, and 10 s at 72 °C for the final extension. The synthesized oligonucleotide primers used for the tested genes are listed in Table 1.

**Table 1:** qPCR primers used in the gene expression analysis.

Gene	GenBank accession no	Sense 5–3	Antisense 5–3	Product size (bp)
*Β-actin*	(NM_007393)	CTGTCCCTGTATGCCTCTG	TTGATGTCACGCACGATT	221
*Nrf2*	NM_031789.2)	TCCATTTCCGAGTCACTGAACCCA	TGACTCTGACTCTGGCATTTCACT	177
*HO-1*	(NM_010442)	GACAGAAGAGGCTAAGACCGC	TCCAGACGTTTCTTCCATCC	213

Using the 2^−ΔΔCt^ method, relative quantification of gene expression was calculated. Gene expression was normalized to *β-actin* (Herath et al. 2020). qPCR reactions for each primer set were repeated three times to verify the reproducibility of results.

### Enzyme-linked immunosorbent assay (ELISA)

Immunoreactivity of AMPK, PI3K, AKT, Nrf2, HO-1, IL-1β, and TNF-α was measured in kidney tissue with ELISA kit (Koma, biotech, Korea) instructions. Optical densities were calculated using an ELISA reader (DAS company, Rome, Italy) at 450 nm and they were expressed as Pg/ml.

### Data and statistical analysis

Data were analyzed using statistical software Prism version 8.0 (GraphPad, San Diego, CA). Normality of the all data was performed with Shapiro–Wilk normality test. Data have a normal distribution (Shapiro–Wilk test, *p* ≥ 0.05) and were analyzed by parametric test one-way analysis of variance ANOVA (post hoc Tukey's HSD) for multiple comparisons. All results were expressed as means ± SD for each group. Non parametric analysis was used in histological score and was analyzed by Mann–Whitney nonparametric *U* test for comparing two groups. Values of *P* < 0.05 were regarded statistically significant.

## Results

### Characterization of PEG-AuNPs

**Fig. 1:**
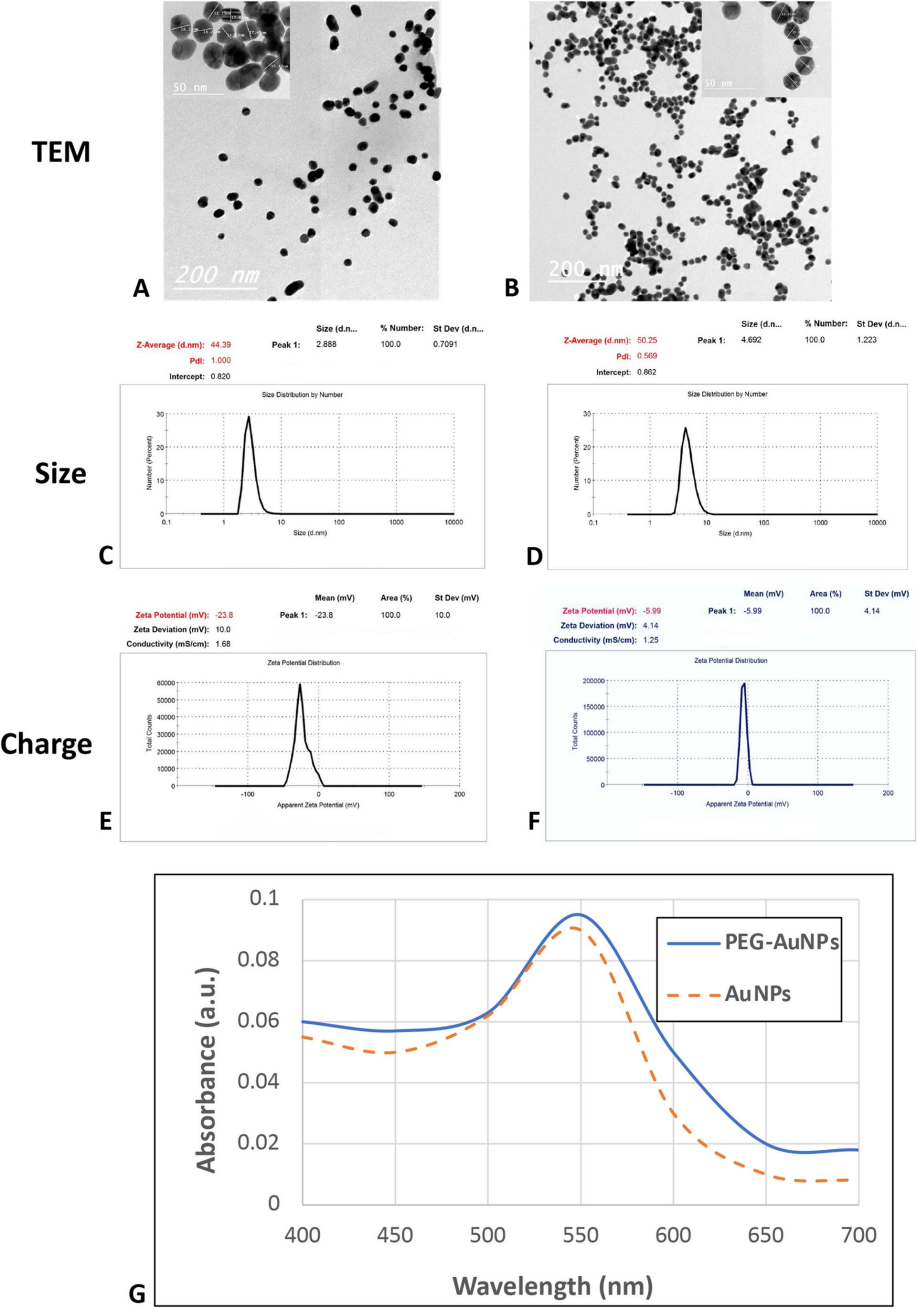
Characterization of gold nanoparticles. TEM micrographs (**A**, **B**) showed the particle of naked AuNPs and PEG-AuNPs. **C** and **D** showed the dynamic light scattering (DLS) of AuNPs and PEG-AuNPs, respectively, the zeta potential of AuNPs and PEG-AuNPs (**E**, **F**), the absorption of the AuNPs and PEG-AuNPs (**G**)

The surface of AuNPs was modified by using a capping agent as polyethylene glycol (PEG) to improve the biological response. The AuNP concentration in 1 mL was measured by an inductively coupled plasma mass spectrometry and displayed 0.125 mg/ml. AuNPs and PEG-AuNPs were characterized using a transmission electron microscope and dynamic light scattering (DLS) for both samples. TEM images of both synthesized naked AuNPs and PEG-coated AuNPs (Fig. 1A, B) showed that the average particle sizes were mainly round and spherical with a diameter ranged between 16 and 25 nm without noticeable aggregations. DLS results showed the increment of hydrodynamic diameter of the nanoparticles coated with the PEG in Fig. 1D in comparison with naked AuNPs (Fig. 1C). The zeta potential of AuNPs and PEG-AuNPs in normal saline was found to be − 23.8 mV and − 5.99 mV, respectively (Fig. 1E, F). A change in the absorption of the PEGylated gold nanoparticles at a longer wavelength was observed in Fig. 1G.

### Glucose level, body and kidney weight

As shown in Table 2, the streptozotocin + I/R-treated mice DI/R group revealed the most common symptoms of diabetes, including hyperglycemia and weight loss in comparison to the non-diabetic group NS (vehicle-treated mice). Our result shows that the low dose (40 µg/kg) of PEG-AuNPs did not cause an obvious change in the glucose level and the body and kidney weights. The glucose level and kidney weight decreased significantly in the PEG-AuNPs 150 µg/kg; the significant decrease (*P* < 0.05) was observed in the 400 µg/kg PEG-AuNPs dose compared to DI/R group. The body weight increased gradually with PEG-AuNPs 150 µg/kg and exhibited an increased value in the dose of 400 µg/kg.

**Table 2:** Effects of PEG-AuNPs on glucose concentration, body, and kidney weight

	NS	DS	DI/R	DI/R PEG-AuNPs 40 µg/kg	DI/R PEG-AuNPs 150 µg/kg	DI/R PEG-AuNPs 400 µg/kg
Glucose (mmol/liter)	9.25 ± 1.25	19.55 ± 0.95*	25.45 ± 1.55^*#^	23.45 ± 2.44^*#^	18.34 ± 1.98^**α**^	12.45 ± 1.95^**α**^
Body weight (g)	26.52 ± 2.09	20.55 ± 0.73*	17.45 ± 2.55^*#^	19.47 ± 1.95^*^	20.55 ± 1.45^**α**^	22.78 ± 2.33^**α**^
Kidney weight (g)	0.51 ± 0.25	0.63 ± 0.35*	0.79 ± 0.29*^#^	0.70 ± 0.65^*^	0.62 ± 0.14^**α**^	0.59 ± 0.54^**α**^

Data are expressed as mean ± SD (*n* = 12). *P < 0.05 vs. NS ^#^*P* < 0.05 vs. DS and ^α^*P* < 0.05 vs. DI/R group. *NS*, normal sham; *DS*, diabetic sham; *DI/R*, diabetic ischemia/reperfusion; *AuNPs*, gold nanoparticles; *PEG*, polyethylene glycol.

### Effect of PEG-AuNPs on the renal histopathological damage

**Fig. 2:**
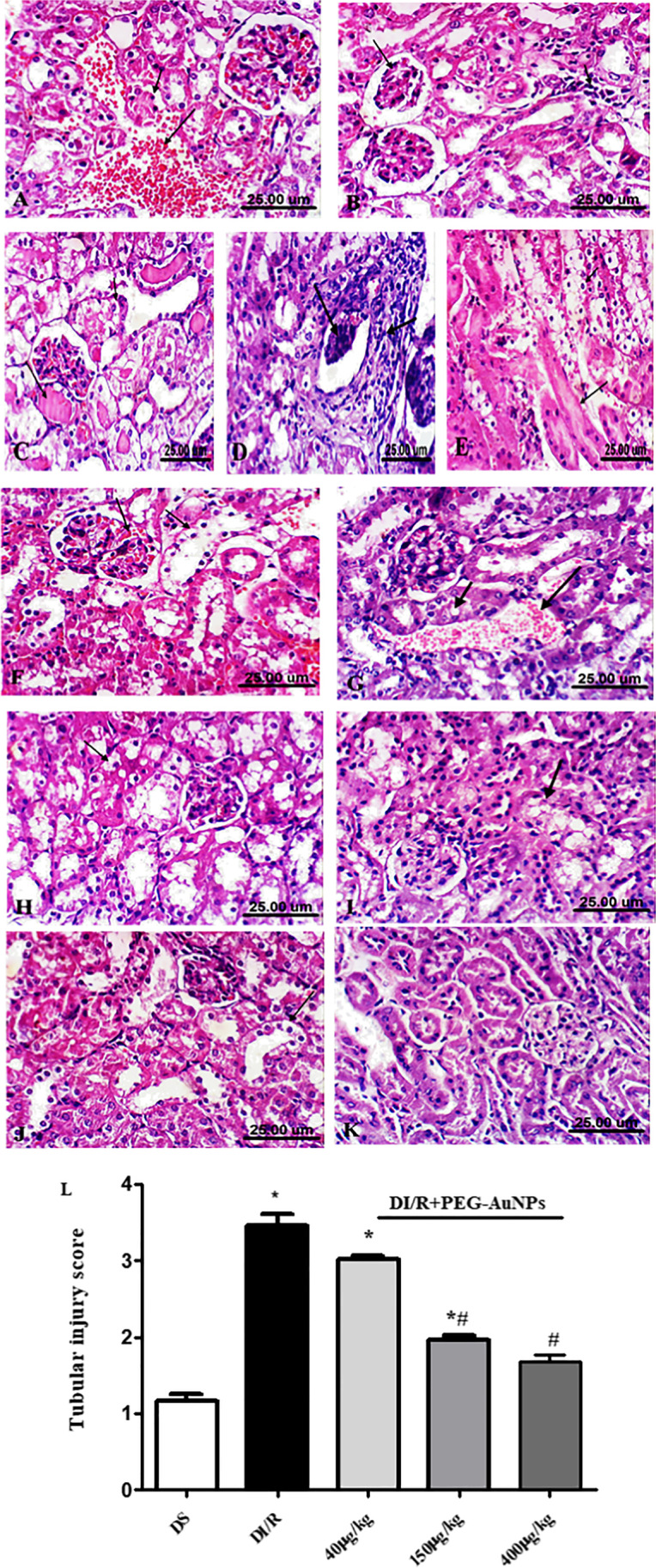
Photomicrographs of kidney sections of mice (hematoxylin and eosin staining, scale bar 25 µm). The diabetic sham (DS) group (**A**, **B**) showing vacuolar degeneration of renal tubular epithelium, focal renal hemorrhage and congestion of glomerulus, interstitial inflammatory cell infiltration, and slight atrophy of glomerular tuft. The diabetic renal ischemia–reperfusion (DI/R) group (**C**–**E**). Micrograph (**C**) showing vacuolar degeneration of renal tubular epithelium and proteinaceous cast in the lumen of renal tubules, **D** showing periglomerular fibrosis associated with inflammatory cell infiltration and atrophy of glomerular tuft, while **E** showing vacuolar degeneration of epithelium of renal tubules accompanied with coagulative necrosis of focal renal tubules. The DI/R + PEG-AuNPs 40 µg/kg group (**F**, **G**) showing cytoplasmic vacuolization of epithelial cells lining some renal tubules, slight congestion of glomerular tuft, and congestion of renal blood vessel. The DI/R + PEG-AuNPs 150 µg/kg group (**H**, **I**). Micrograph (**H**) showing vacuolar degeneration of renal tubular epithelium, whereas **I** showing necrobiosis of epithelial lining renal tubules. The DI/R + PEG-AuNPs 400 µg/kg group (**J**, **K**). Micrograph (**J**) showing slight cytoplasmic vacuolization of epithelial lining some renal tubules, while **K** showing normal renal parenchyma. **L** Bar graph of tubular injury score. Data are presented as mean ± SD (*n* = 12). *P < 0.05 vs. DS;.^#^*P* < 0.05 vs. DI/R group

The typical renal tubular damage includes severe vacuolar degeneration, loss of brush border, necrosis of focal renal tubules, and focal renal hemorrhage. The comparison between the five groups can be distinguished histologically in Fig. 2A–K affirmed by the histological damage score of renal tissue (Fig. 2L) and the renal function (Fig. 4). The diabetic renal I/R group was the most affected group where renal tubular epithelia exhibit vacuolar degeneration accompanied with coagulative necrosis of some renal tubules and appearance of the proteinaceous cast in the lumen of others; the periglomerular tissue exhibits fibrosis linked with inflammatory cell infiltration and atrophy of glomerular tuft. The diabetic I/R mice injected with PEG-AuNPs 150 µg/kg revealed necrobiosis of some epithelial lining renal tubules and exhibited less tubular atrophy and interstitial fibrosis. The DI/R PEG-AuNPs 400 µg/kg group showed slight cytoplasm vacuolization of epithelial lining renal tubules. This suggested that PEG-AuNPs attenuated the effect of the renal I/R in diabetic mice which was confirmed by the kidney function examination.

### Effect of PEG-AuNPs on the renal fibrosis in I/R injury in diabetic mice

**Fig. 3:**
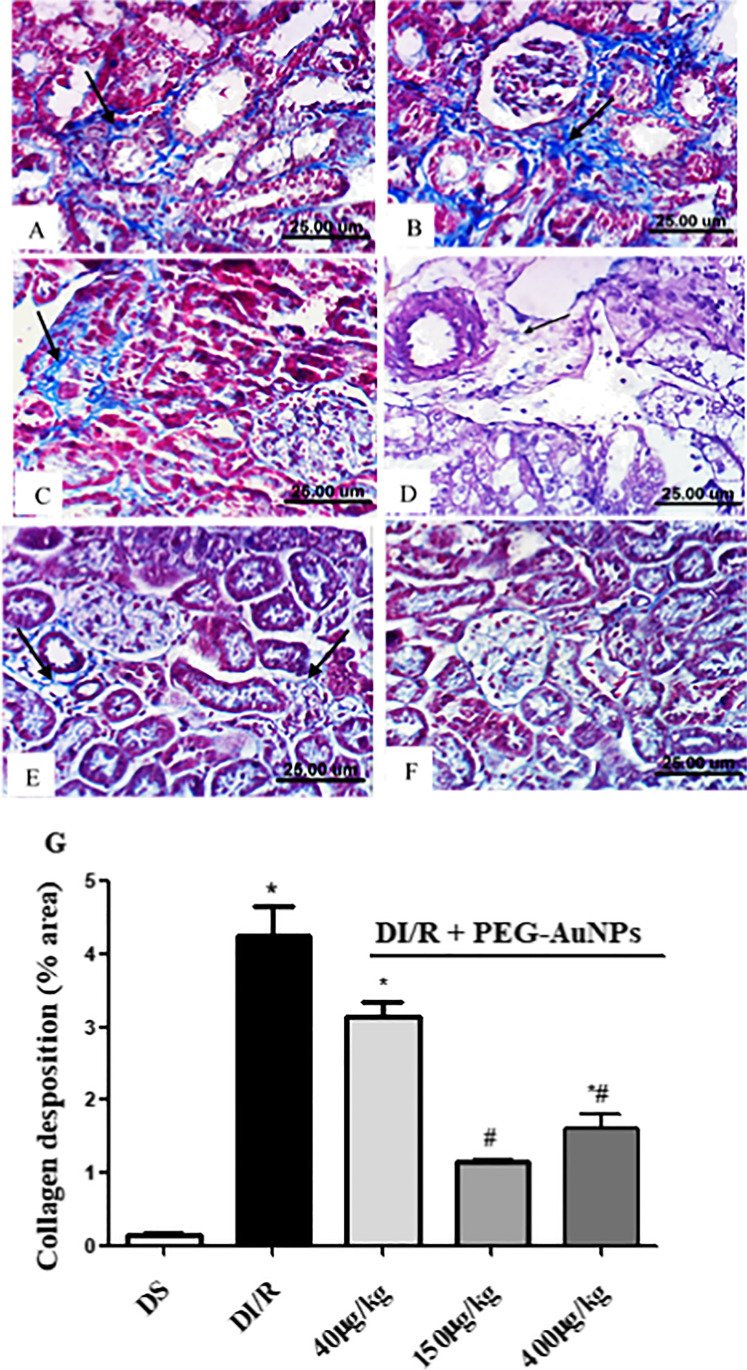
Photomicrographs of kidney sections of mice (Masson’s trichrome stain, scale bar 25 µm). The diabetic sham (DS) group (**A**) showing a moderate positive histochemical reaction for collagen fibers. The DI/R group (**B**) showing a strong positive histochemical reaction for collagen fibers. The DI/R + PEG-AuNPs 40 µg/kg group (**C**, **D**). Micrograph (**C**) showing a moderate positive histochemical reaction for collagen fibers, while **D** showing a weak positive histochemical reaction for collagen fibers. The DI/R + PEG-AuNPs 150 µg/kg group (**E**) showing a weak positive histochemical reaction for scanty collagen fibers. The DI/R + PEG-AuNPs 400 µg/kg group (**F**) showing poor histochemical reaction for collagen fibers. **G** Collagen deposition % area. DI/R causes more collagen deposition compared with other groups. Data are expressed as the mean ± SD (*n* = 12). ^*^P < 0.05 vs. DS group; ^#^*P* < 0.05 vs. DI/R group

The detection of the collagen disposition in the kidney tissues was evaluated by Masson’s trichrome blue staining and confirmed the massive tubule-interstitial fibrosis. The diabetic sham group DS exhibited a moderate positive histochemical response for collagen fibers in the renal interstitial area and tubular basement membranes, while the diabetic renal DI/R group exhibited the highest collagen deposition in the renal interstitial area, the glomeruli, and the tubular basement membranes compared to the other groups. In the DI/R PEG-AuNPs 40 and 150 µg/kg groups, the collagen fiber was markedly reduced; the glomeruli and tubular basement membranes were stained lighter. Notably, DI/R PEG-AuNPs 400 µg/kg group displayed faint staining of scant collagen fibers (Fig. 3A–F). The intensity of renal fibrosis is illustrated in Fig. 3G

### Effect of PEG-AuNPs on the kidney function

**Fig. 4:**
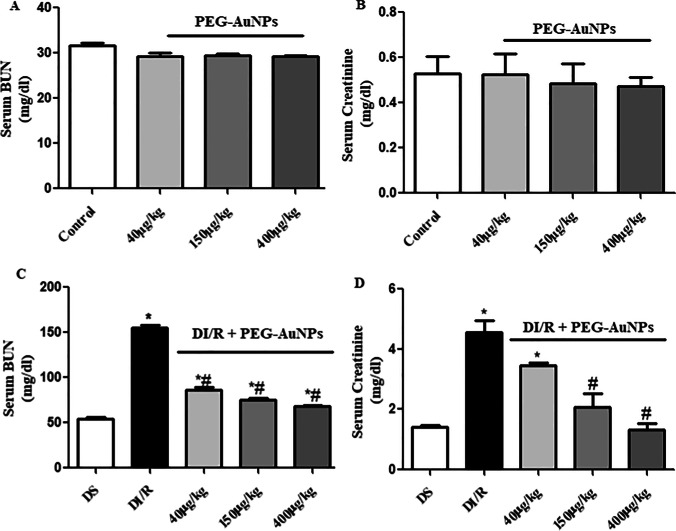
Effect of PEG-AuNPs on serum blood urea nitrogen (BUN) and serum creatinine. **A** BUN and **B** creatinine level of normal mice; **C**, **D** BUN and creatinine of diabetic mice subjected to 30 min ischemia and 48 h reperfusion. Data are presented as the mean ± SD (*n* = 12). ^*^P < 0.05 vs. DS group; ^#^*P* < 0.05 vs. DI/R group

The normal mice were injected with three different doses of PEG-AuNPs and displayed non-significant changes in serum BUN and creatinine (Fig. 4A, B) which reflect the safety of these doses chosen. The levels of BUN and creatinine were elevated significantly (*P* < 0.05) in the diabetic renal ischemia–reperfusion (DI/R) group, indicating impairment in kidney function compared with the DS group and subsequently decreased gradually in the DI/R PEG-AuNPs 40 µg/kg, 150, and 400 µg/kg groups (Fig. 4C, D).

### Kidney ultrastructure examination

**Fig. 5:**
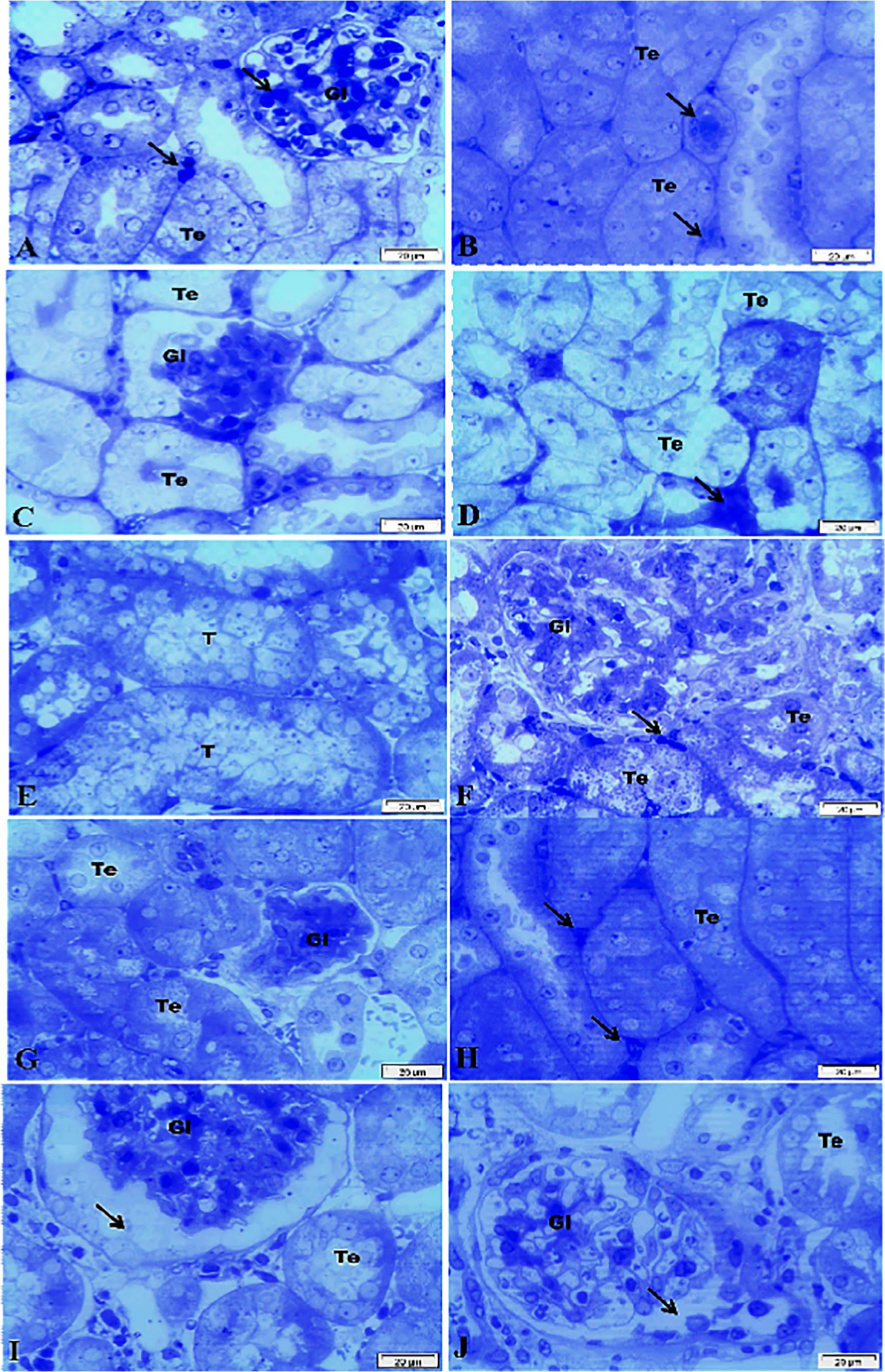
Photomicrographs of kidney semi-thin sections of mice (toluidine blue staining). The diabetic sham (DS) group (**A**, **B**) showing congestion of the interstitial and glomerular, vasculature (arrow) and a variable degree of hydropic, and degeneration of the epithelial cells lining the tubules. The DI/R group (**C**, **D**) showing prominent vacuolar degeneration and ruptured of numerous tubular epithelia, the capillary tufts of the glomeruli compressed with marked dilatation of the urinary space, and the interstitial blood vasculature appeared congested (arrow). The DI/R + PEG-AuNPs 40 µg/kg group (**E**, **F**) showing vacuolation of the most cell lining the tubules with slight congestion of the intratubular vasculature (arrow); the glomeruli showing an increment in the cellularity with narrowing in the urinary space. The DI/R + PEG-AuNPs 150 µg/kg group (**G**, **H**) showing slight congestion of the glomerular and interstitial vasculature (arrows) and slight swelling and vacuolation of the epithelial cell lining tubules. The DI/R + PEG-AuNPs 400 µg/kg group (**I**, **J**) showing swelling and vacuolation of the tubular epithelium with the presence of faintly stained casts in the lumen of some tubules and the parietal epithelial cells of the glomeruli appeared swollen with dilatation of the urinary space (arrow). Abbreviations: Gl, glomeruli; Te, tubular epithelium; T, tubule

Additional description of histological damage appeared in the semi-thin section of the kidney (Fig. 5A–J). The kidney section from the diabetic sham (DS) group shows vasculature and congestion of the tubule interstitial and glomerular, a variable degree of hydropic changes with swelling of the tubular epithelium (Fig. 5A, B). The DI/R group (Fig. 5D) showed the tubular cells become swollen and lightly stained. This group exhibited the greatest extent of damage including tubular vacuolization, degeneration, and rupturing of numerous cells of proximal convoluted tubules; moreover, the lumen of some dilated renal tubules becomes narrow or completely obliterated with thickened basement membranes, the capillary tufts of the glomeruli compressed with marked dilatation of the urinary space, and the interstitial blood vasculature appeared congested. However, PEG-AuNPs 40 µg/kg administration (Fig. 5E, F) was not completely alleviated the vacuolation of neither most of the cell lining the tubules nor the congestion of the intratubular vasculature. The increment in the cellularity of the glomeruli and the narrowing of the urinary space are still present. Figure 5G, H revealed a considerable degree of improvement of kidney tissue in the group of mice treated with PEG-AuNPs 150 µg/kg. These improvements include slight congestion of the glomerular, interstitial vasculature, slight swelling, and vacuolation of the epithelial cell lining tubules. Effect of PEG-AuNPs 400 µg/kg in diabetic ischemic kidney revealed improvements with the appearance of faintly stained casts in the lumen of some tubules, cytoplasmic vacuolization; the parietal epithelial cells of the glomeruli appeared swollen with dilatation of the urinary space (Fig. 5I, J).

### TEM examination of the kidney tissue

**Fig. 6:**
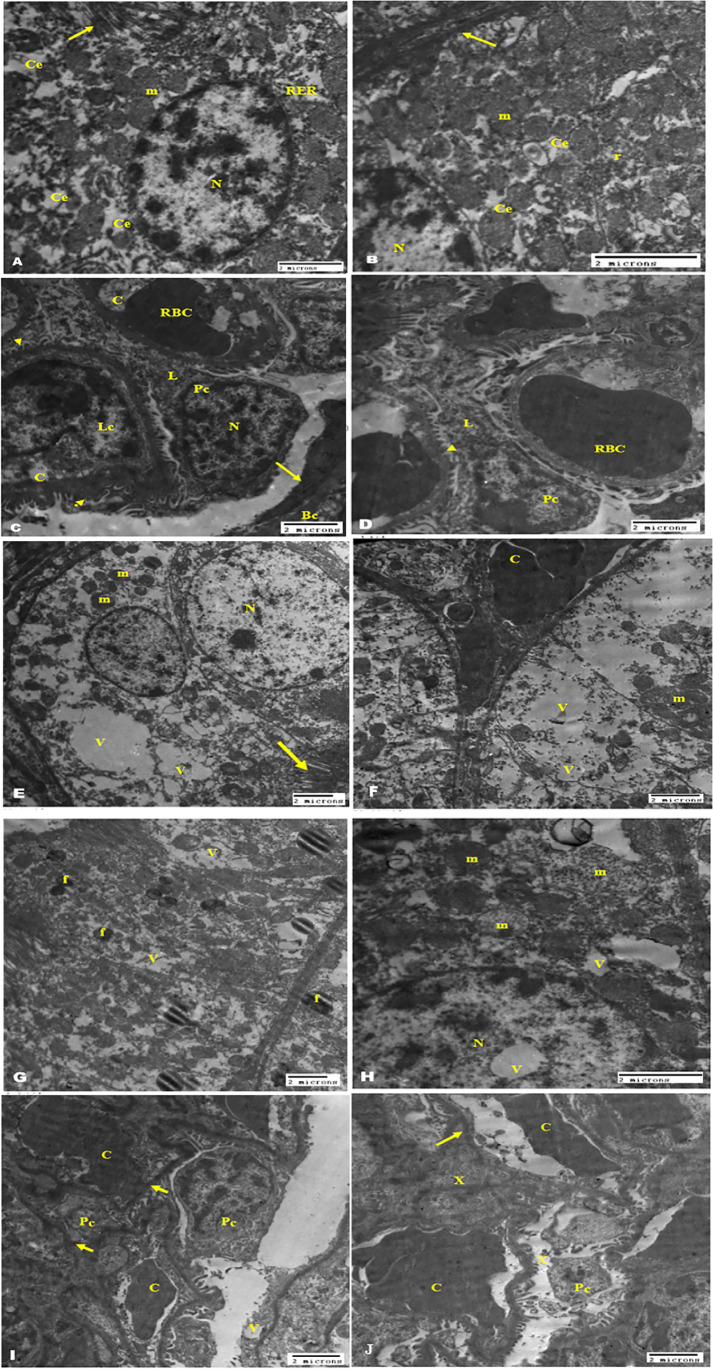
Transmission electron micrographs of the kidney (staining with uranyl acetate and lead citrate). The diabetic sham (DS) group (**A**–**D**). Micrographs (**A**, **B**) showing a tubular epithelial cell with a large and vesicular nucleus, chromatin clumped at the periphery of the nucleus, swollen mitochondria, free ribosome, cytoplasmic edema, and long microvillus (arrow) basement membrane (arrow in micrograph B), while **C** and **D** showing kidney glomerular tissue. Bowman’s capsule with the parietal epithelial cell (arrow) and the visceral epithelial cell or podocytes with a large vesicular nucleus, long primary processes, and short secondary processes (arrowhead) surrounding dilated blood capillaries containing RBCs and leukocytes. The DI/R group (**E**–**J**). Micrographs (**E**–**H**) showing tubular epithelium with vacuolar degeneration, swelling and loss of membranous structure, electron-dense mitochondria, and congestion of the peritubular blood capillaries. Notice the microvilli (arrow), the nucleus contains vacuole in micrograph H and the presence of numerous fat globules in the cytoplasm of the epithelium lining tubules (micrograph G). Micrographs (**I**, **J**) of kidney glomerular tissue showing degeneration and vacuolation of the parietal epithelial cell, congestion of the capillary tufts, atrophy of the podocytes, shrinking of the mesangial matrix (x), and the basement membrane of capillary tufts and parietal cells appeared electron-dense (arrow). Abbreviations: N, nucleus; m, mitochondria; r, ribosome; Ce, cytoplasmic edema; Bc, Bowman’s capsule; Pc, podocytes; L, long primary processes; C, capillaries; RBC, red blood cell; Lc, leucocytes; RER, rough endoplasmic reticulum; V, vacuole; f, fat globules

**Fig. 7:**
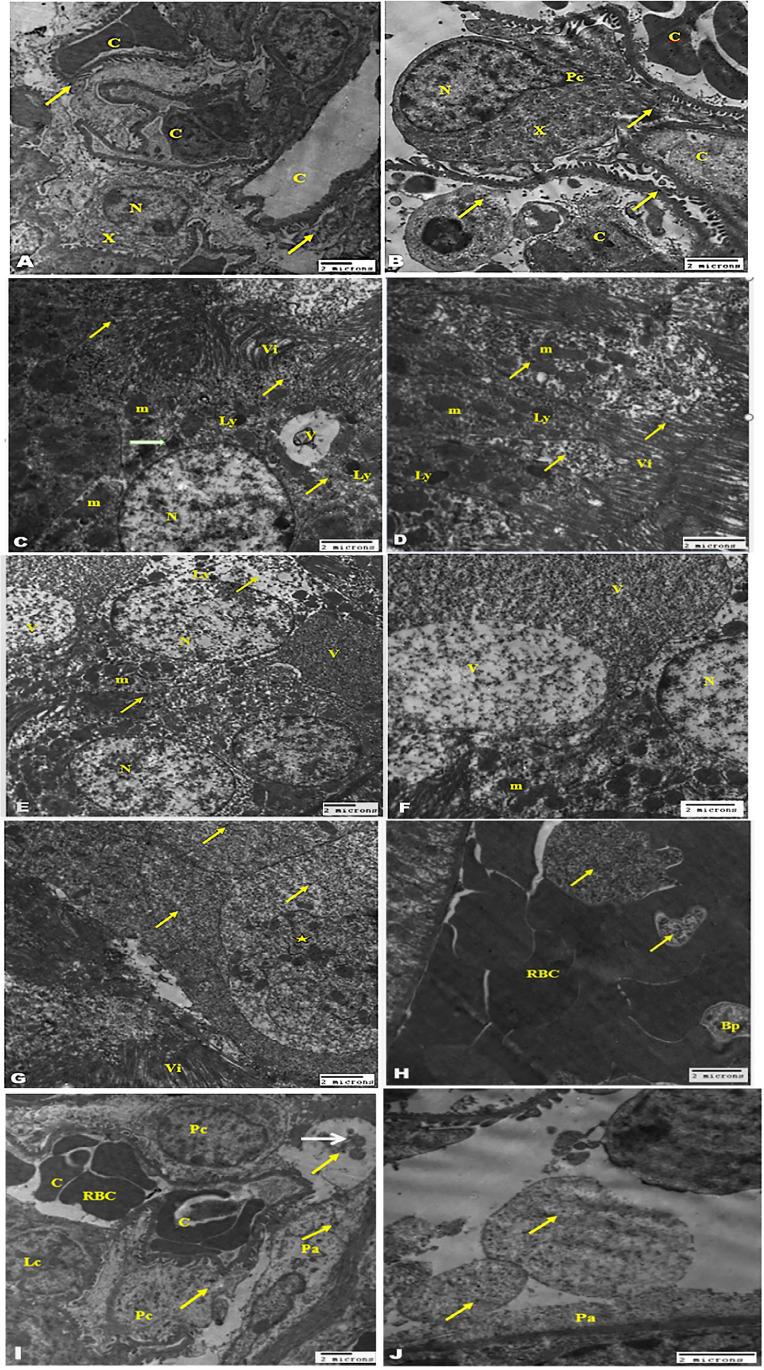
Transmission electron micrographs of the kidney (staining with uranyl acetate and lead citrate). The DI/R PEG-AuNPs 40 µg/kg group (**A**–**F**). Micrographs (**A**, **B**) showing tubular lining epithelial cells contain a vesicular nucleus, cytoplasm having mitochondria variable in size and electron density, numerous variable size membranous vacuoles that contain fine electro dense nano-particles, and electron-dense lysosomes (arrow). Notice the presence of a large amount of variable size small membranous vesicles (star). Micrograph (**D**) of kidney tubules showing congestion of the peritubular capillary and the microvilli in the luminal surface. While **E**, **F** micrographs of kidney glomerular tissue showing swelling and vacuolation of the parietal cell of the capsules, podocytes, mesangial cell, dilated blood capillaries, and the presence of electron-dense nano-particles in the urinary space (arrow). The DI/R PEG-AuNPs 150 µg/kg group (**G**–**J**) showing the cell lining of proximal and distal convoluted tubules nearly of normal morphological appearance of nucleus and mitochondria; the surface microvillus and few variable size membrane-bounded vacuoles contain electron dense nano-particles. Notice the presence of renal casts in the proximal tubules (star) and the electron-dense particles in the cell cytoplasm mostly near the surface (arrows). Abbreviations: N, nucleus; m, mitochondria; Pa, parietal cell; Pc, podocytes; MS, mesangial cell; C, capillaries; Lu, lumen; V, vacuole; U, urinary space; Vi, microvilli

**Fig. 8:**
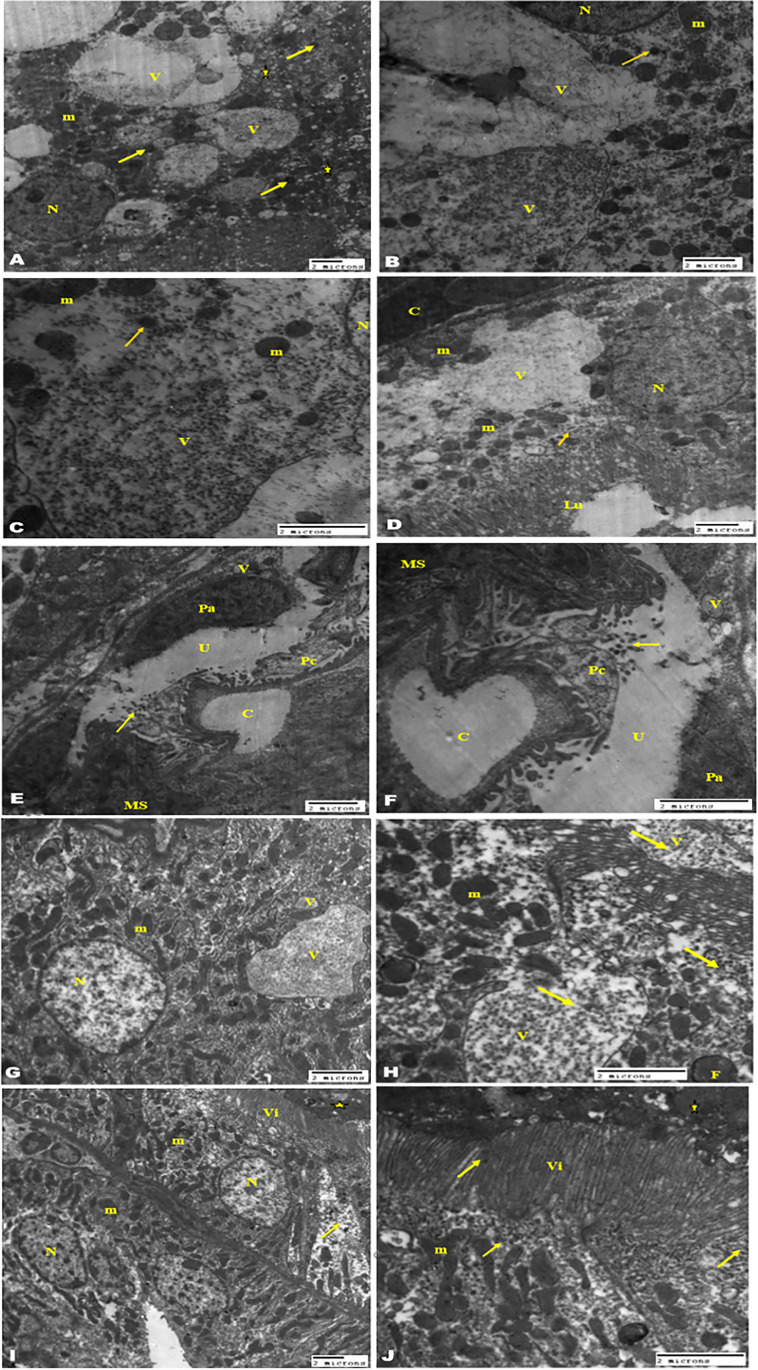
Transmission electron micrographs of the kidney. The DI/R PEG-AuNPs 150 µg/kg group (**A**, **B**) showing the glomerular tissue with capillary tufts containing blood cells; large podocytes have a vesicular nucleus, primary and secondary processes, and cytoplasm rich with cell organelles (X). Notice the presence of few electron-dense nano-particles (arrows). The DI/R PEG-AuNPs 400 µg/kg group (**C**–**J**). Micrographs (**C**–**G**) showing tubular lining epithelial cells having large vesicular nucleus and cytoplasm contains mitochondria mostly condensed, vacuoles contain electron-dense nanoparticles and membranous depress, and numerous electron-dense lysosomes some contain nanoparticle (white arrow). Micrograph (G) showing membrane-bounded bodies filled with nanoparticles (arrows) and some of them contain cellular depress (star). Notice the presence of long microvilli in the luminal surface. Micrograph (**H**) of the interstitial blood vessel of a kidney showing congestion contains RBCs and blood platelets and membrane-bounded vacuoles contain nanoparticles (arrows). Micrographs (**I**, **J**) of kidney glomeruli of this group showing the parietal cells and the visceral or podocytes having light electron-dense cytoplasm contain electron-dense nanoparticles; the blood capillary tufts congested contained RBC and leukocytes. Notice the urinary space contains portentous material (white arrow) and electron-dense nanoparticles (yellow arrows). Abbreviations: N, nucleus; m, mitochondria; Ly, lysosomes; Pa, parietal cell; Pc, podocytes; C, capillaries; V, vacuole; Vi, microvilli; RBC, red blood cell; Bp, blood platelets; Lc, leukocytes

TEM examination of the kidney from the diabetic group exhibited milled histopathological changes including vacuolar degeneration of the tubular epithelium with swelling of the mitochondria and clumping of the nuclear chromatin of their nucleus and congestion of the stromal and glomerular capillary tufts (Fig. 6A–D). The ischemic kidney of the diabetic group (Fig. 6E–J) revealed severe vacuolation with the presence of fat globules and rupture of the most tubular epithelium; the cytoplasm showed some amorphous density mitochondria with destroyed cristae with severe congestion of the interstitial blood vasculature. The micrographs showed also dilatation of the urinary space, compression of the glomerular tufts atrophy of the podocytes, and shrinking of the mesangial matrix. The micrographs of kidney mice treated with 40 µg/kg PEG-AuNPs (Fig. 7A–F) showed limited improvement and the pathological changes slightly decreased than that observed in the ischemic group. The tubular epithelium showed presences of numerous membrane-bounded vacuoles that contain nano-gold particles, electron-dense mitochondria, and lysosomes. The glomeruli showed congestion, swelling, and vacuolation of the podocytes and the parietal cell of the capsules. We noticed the dilated blood capillaries as well and the appearance of electron-dense nano-particles in the urinary space. Effect of 150 µg/kg PEG-AuNPs in diabetic ischemic kidney revealed a considerable degree of improvement of kidney tissue with the presence of renal casts in the proximal tubules and few vacuoles contain nano-gold in the lining tubular epithelium (Figs. 7G–J and 8A, B). The group of mice treated with 400 µg/kg nano-gold particles exhibited improvements with the appearance of cytopathic changes in both tubules and glomeruli. These changes included the presence of membrane-bounded vacuoles that contain nano-gold particles; these vacuoles also exist in the blood vessels and the lumen of the tubules. The epithelial cells of the glomerular tissue showed vacuolation, and the parietal cells and the visceral or podocytes appeared swollen with light electron-dense cytoplasm that contains electron-dense nanoparticles (Fig. 8C–J). This ultrastructure examination suggested that PEG-AuNPs (mainly150 and 400 µg/kg) reduced the destructive effect of the renal ischemia–reperfusion in diabetic mice.

### PEG-AuNPs reduced MDA and enhanced the antioxidative activity following renal I/R injury in diabetic mice

**Fig. 9:**
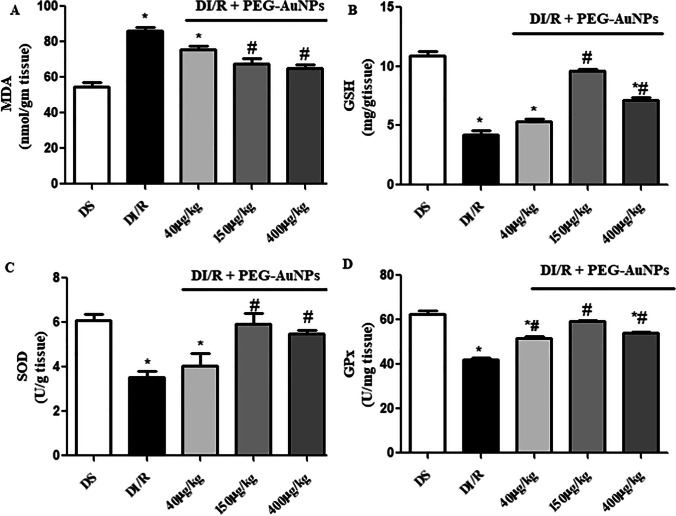
Effect of PEG-AuNPs on MDA and antioxidant enzymes following renal I/R in diabetic mice. Malondialdehyde (MDA) (**A**); glutathione reduced GSH (**B**); superoxide dismutase (SOD) (**C**); glutathione peroxidase (GPx) (**D**); data are presented as the mean ± SD (*n* = 12). ^*^P < 0.05 vs. DS group; ^#^*P* < 0.05 vs. DI/R group

The results displayed that the level of MDA elevated significantly (*P* < 0.05) in the kidney of the diabetic renal ischemia–reperfusion (DI/R) group as compared with the diabetic sham group. Administration of PEG-AuNPs 150 and 400 µg/kg decreased significantly (*P* < 0.05) the MDA level compared with the DI/R group as shown in Fig. 9A. Intravenous injection of coated AuNPs 40 and 400 µg/kg increased the concentrations of GSH as well as the activities of SOD and GPx enzymes compared with the diabetic renal ischemia–reperfusion (DI/R) group. The results in Fig. 9B–D exposed that the level of GSH and the activities of SOD and GPx enzymes increased significantly (*P* < 0.05) in PEG-AuNPs 150 µg/kg compared with the DI/R group.

### Effect of PEG-AuNPs on the expression of Nrf2 and HO-1 during renal I/R in diabetic mice

**Fig. 10:**
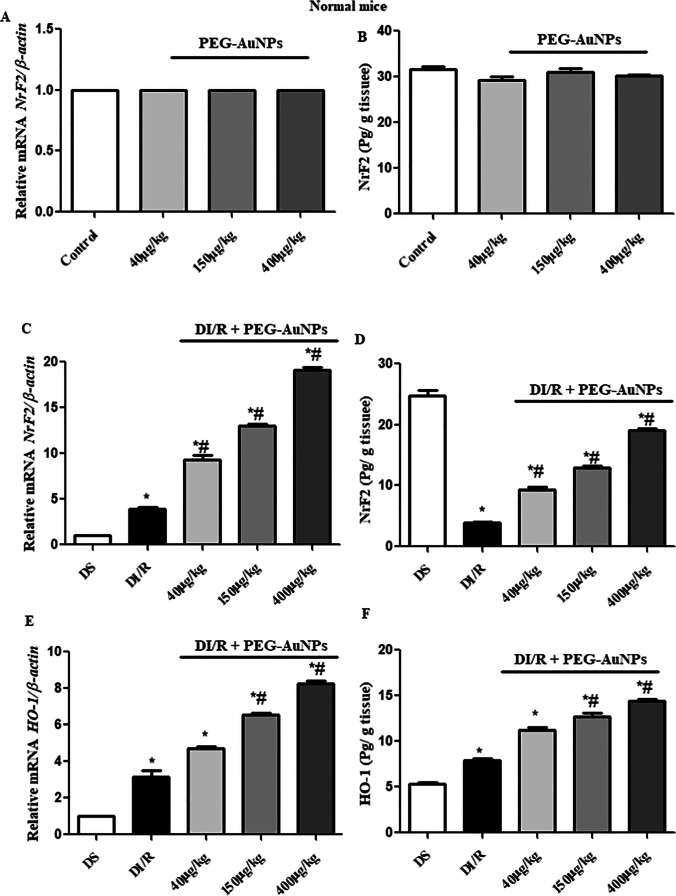
PEG-AuNPs upregulate the kidney expression of Nrf2 and HO-1. qRT-PCR of Nrf2 and HO-1 (**A**, **C**, and **E**). Protein concentration of Nrf2 and Ho-1 was measured by ELISA (**B**, **D**, and **F**). Data are presented as the mean ± SD (*n* = 12). ^*^P < 0.05 vs. DS group; ^#^*P* < 0.05 vs. DI/R group

To explore if PEG-AuNPs of different concentrations cause any deleterious effect on the antioxidant response in renal tissues, we tested the three different doses of PEG-AuNPs on the normal mice. Figure 10A, B revealed no change in the gene expression and protein concentration of Nrf2. To investigate the controlling mechanism of PEG-AuNPs in renal I/R injury, we used qRT-PCR to detect the changes in gene expression of Nrf2 and its downstream factors. Following intravenous administration of different doses of PEG-AuNPs, the expression of Nrf2 and HO-1 increased significantly (*P* < 0.05) in the groups treated with PEG-AuNPs (150 and 400 µg/kg) (Fig. 10C, E). Protein levels measured by ELISA of Nrf2 and HO-1 were markedly reduced under diabetic renal I/R, whereas the supplement of PEG-AuNPs reversed these decreases (Fig. 10D, F). However, PEG-AuNPs treatment activated Nrf2 signaling to ameliorate the renal injury triggered by diabetic I/R.

### Effect of PEG-AuNPs on the expression of phosphorylated AMPK in the renal tissue

**Fig. 11:**
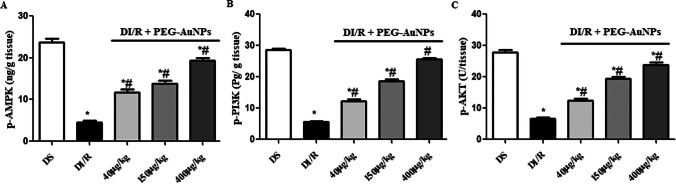
PEG-AuNPs activate the AMPK and PI3K/AKT signaling pathway. **A** p-AMPK, **B** p-PI3K, and **C** p-AKT protein levels measured by ELISA. Data are presented as the mean ± SD (*n* = 12). ^*^P < 0.05 vs. DS group; ^#^*P* < 0.05 vs. DI/R-group

To examine the cellular mechanisms mediating the effects of PEG-AuNPs on diabetic I/R injury, we measured the phosphorylation level of AMPK, PI3K, and AKT in the kidneys by ELISA. After 48-h reperfusion, renal tissues showed a severe decline in AMPK, PI3K, and AKT expression as compared to the sham-operated group. Whereas PEG-AuNPs at the highest treatment dose (400 µg/kg) increased significantly (*P* < 0.05) the concentration of AMPK, PI3K, and AKT compared to the DI/R group (Fig. 11A, B, and C).

### Effect of PEG-AuNPs on the proinflammatory cytokines

**Fig. 12:**
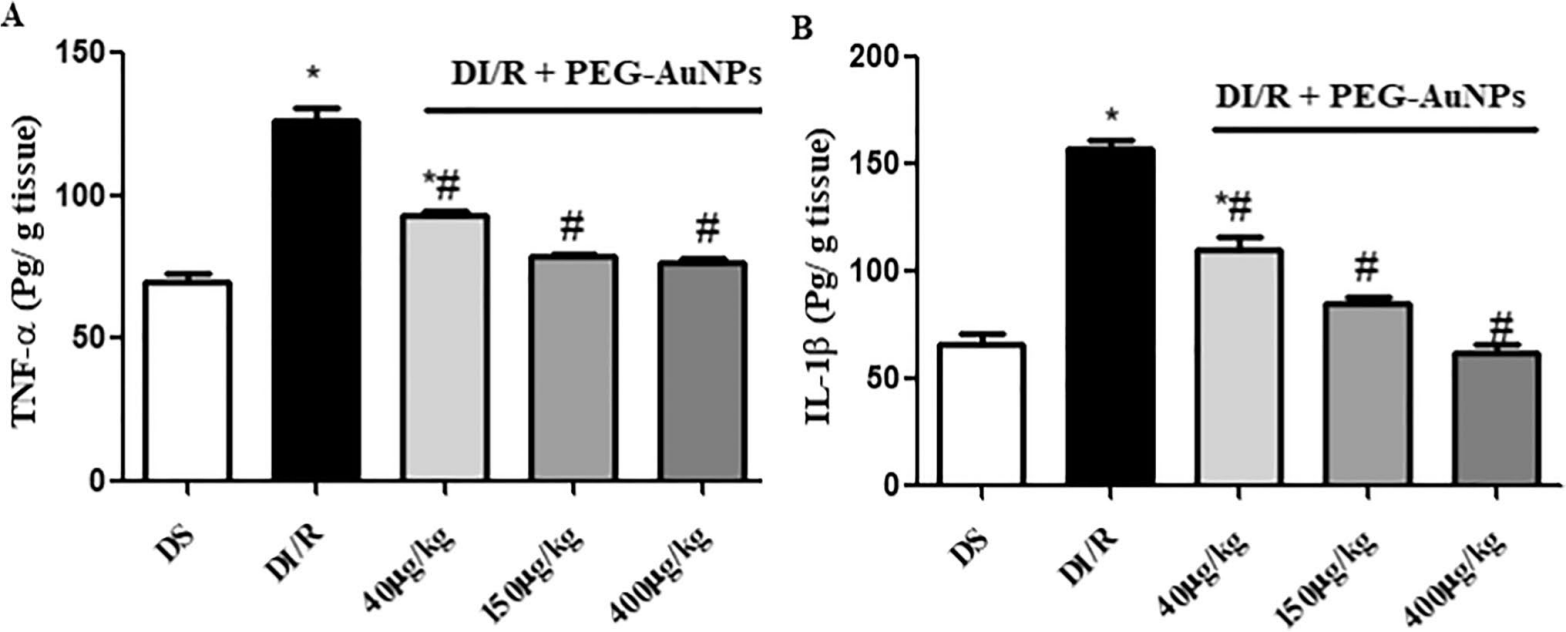
Effect of PEG-AuNPs on proinflammatory cytokines, tumor necrosis factor-alpha (TNF-α) (**A**), and interleukin-1 beta (IL-1β) (**B**) after renal I/RI in diabetic mice. The concentration of cytokines was measured by ELISA. Data are presented as the mean ± SD (*n* = 12). ^*^P < 0.05 vs. DS group; ^#^*P* < 0.05 vs. DI/R group

The protein level of TNF-α and IL-1β was significantly greater in the DI/R group than those in the diabetic sham group. However, the treatment with PEG-AuNPs could significantly reduce the protein concentration of TNF-α and IL-1β after renal I/RI in diabetic mice (Fig. 12A, B).

## Discussion

In this study, we proved that PEG-AuNPs exert protective effects against renal I/R injury in diabetic mice–induced AKI through inhibiting oxidative stress and inflammatory responses, which were mostly reliant on the enhancement of the Nrf2 pathway via activation of AMPK.

Diabetes mellitus patients have reported increased susceptibility of the kidney to AKI and chronic kidney diseases (CKD) (Mejía-Vilet et al. 2007). Renal damage following I/R injury is a main cause of AKI in animal models of type 1 and type 2 diabetes (Melin et al. 2002; Peng et al. 2015). A strong correlation exists between glycemic and the incidence and progression of microvascular complications (Lepore et al. 2002). A 25-min period for ischemia has been applied in several studies to cause mild renal injury (Park 2004). The reperfusion of blood flow abruptly into ischemic tissue may develop more damage that is not present at the ending of ischemia (Zhao 2010). The prolonged ischemia–reperfusion injury may induce cell damage and cause apoptosis, necrosis, and autophagy (Zhu et al. 2016; Ling et al. 2017). Although several studies have been conducted, the accurate mechanism of induced renal injury remains incompletely understood. Recent studies believe that some pathologic processes play important roles in I/R-induced renal injury. These processes include the exacerbate inflammatory response, the microcirculatory disorders, the release of ROS (Hong et al. 2017), the mitochondrial dysfunction, the endoplasmic reticulum stress, the loss of kidney architecture, and finally the renal dysfunction (Galvan et al. 2017; Humphreys 2018, Lv et al. 2018). Until now, there is no effective therapy applied for the cure of renal I/RI; patients remain to receive treatment for the attenuation of renal I/R injury that includes various antioxidant and anti-inflammatory drugs, erythropoietin, endocrine hormones, small interfering RNA, and others (Malek and Nematbakhsh 2015), but several problems have avoided their clinical application. Thus, it is important to explore effective treatment to decrease renal I/R injury**.**

Gold nanoparticles (AuNPs) have been widely used for nearly all biomedical applications such as prevention, diagnosis, and therapy (Khan and Khan 2018). Their biologically relevant size, non-toxic nature, high surface area, and ability to easily functionalize with biomolecules, as well as their optical characteristics, make them ultimate for medical applications (Kong et al. 2017). Additionally, low-cost preparation techniques, easy synthesis, and their high stability in the biological environment add more credit for their extensive applications mainly towards humans (Si et al. 2017). Recently, Justo et al. proved that PEGylated gold nanoparticles have potential use in different medical applications such as gene therapy and drug delivery systems (Zamora-Justo et al. 2019). In this study, we modify the surface of naked AuNPs by coating them using polyethylene glycol (PEG) as a spacer. This surface modification can alter the chemical and physical features of nanoparticles including the following: change the hydrophilic and hydrophobic properties, reduce surface charges, and create new functions for nanoparticles, such as membrane permeability and targeting (Lipka et al. 2010; Jiao et al. 2020). Moreover, PEG improves the focalization of nanogold particles (Remant Bahadur et al. 2006) and increases the blood circulation lifetime of NPs (Rana et al. 2012). Zhang (Zhang et al. 2011) also revealed that PEG coating gold nanoparticles might modulate the endocytotic uptake and cellular trafficking and stimulate the anti-PEG antibody secretion that may accelerate the blood clearance (Yang et al. 2014). In addition, PEG is used as a surface-modifying substance due to its strong ability to prevent protein adhesion in the bloodstream and further to avoid immune cell recognition (Guo et al. 2013). All the mice in the current study were injected intravenously with round and spherical PEG-AuNPs with a diameter ranging between 16 and 25 nm; the size used appeared to be nontoxic. This result was similar to those previously reported by Pan et al. (Pan et al. 2007) who declared that the AuNPs larger than 15 nm are comparatively nontoxic. Also, Connor et al. (Connor et al. 2005) found that AuNPs of about 18 nm in diameter might penetrate the cells without cell injury and toxicity. Zheng et al. (Zheng et al. 2019) established that 20-nm AuNPs increased cell viability, improved oxidative stress and apoptosis, and alleviated mitochondrial respiration. The doses of PEG-AuNPs used in our study were 40, 150, and 400 µg/kg supported by previous literature (Lasagna-Reeves et al. 2010; Zhang et al. 2010). These studies showed that the low concentrations of AuNPs did not cause any noticeable toxicity. Lasagna-Reeves declared that the use of dose range 320–3200 µg/kg/day has no harmful effect on different biochemical parameters (Lasagna-Reeves et al. 2010). Most of the nanoparticles enter cells by endocytosis (Chithrani and Chan 2007), phagocytosis (Zheng et al. 2019), transmembrane channels, and adhesive interactions (De Jong et al. 2008) or bound to cell surface receptors (De Jong 2008).

In our study, the renal tissues stained with hematoxylin and eosin affirmed by the histological damage score showed the harmful effect of I/R on the renal tissues of diabetic mice. The tissues that exhibited inclusive tubule injuries such as cytoplasmic vacuolation, tubular dilation, and hyaline casts were revealed in the lumen of some tubules; these results are consistent with Sadek et al. (2013) and Yang et al. (2019). The periglomerular tissue exhibits fibrosis connected with inflammatory cell infiltration, and atrophy of glomerular tuft deformation of renal corpuscles was noted, in the form of shrunken glomeruli as declared by (Sadek et al. 2013). Moreover, during the period of hypoxia in I/R, the activated myofibroblasts promote the loss of capillaries and are considered as a source of renal inflammation and fibrosis (Portilla 2014). These findings are consistent with our results in Masson’s trichrome staining that was used in this study to demonstrate renal fibrosis followed by renal I/R. Diabetic renal I/R showed more collagen deposition in the renal interstitial area; the glomeruli and the tubular basement membranes confirmed the massive tubule-interstitial fibrosis; these results are in agreement with (Wu et al. 2013).

The ischemic kidney of the diabetic group in our ultra-structure study revealed severe deformation in most of the tubular epithelium, diminution in the brush border of tubules, and congestion of peritubular capillary. These results are consistent with a previous report showing that the I/R group suffered from significant tubular necrosis, congestion and medullary hemorrhage (Chen et al. 2015), loss of brush border, cast formation, tubular dilatation (Hatcher et al. 2015; Han et al. 2017), and leukocytes activation (Đurašević et al. 2019). Galluzzi et al. declared that renal tubular cells express cell surface death receptors (TNF-α) that upon activation during renal ischemia activate caspases and initiate apoptosis, which contributes to AKI (Galluzzi et al. 2012). In addition, our TEM results showed that in diabetic mice and after I/R injury mitochondria, more severe ultrastructural changes such as mitochondrial swelling, vacuolization in the matrix, and loss of cristae are shown. Mitochondrial fragmentation and dysfunction were observed in renal ischemia following ATP depletion (Portilla 2014). Eltzschig et al. affirmed that subsequent mitochondrial dysfunction may lead to tubular epithelium apoptosis and loss of function (Eltzschig and Eckle 2011). The DI/R group showed also dilatation of the urinary space, compression of the glomerular tufts atrophy of the podocytes, and shrinking of the mesangial matrix. These findings were similar to those earlier reported by Sadek et al. (2013) and Xie et al. (2017) who marked the destruction of renal corpuscles and the glomerular damage and noted the loss of almost all glomerular tufts. Han et al. (2017) found that untreated I/RI mice displayed severe expansion of Bowman’s capsule and interstitial edema 48 h after renal I/RI. Glomeruli remain vulnerable to further injury by interstitial fibrosis even after recovery from acute kidney injury (Park et al. 2018). The TEM examination showed limited alleviation in the renal ischemic tissues in mice injected with 40 µg/kg PEG-AuNPs, while the groups of mice treated with 150 and 400 µg/kg PEG-AuNPs respectively exhibited a considerable degree of improvement of kidney tissue. These results proposed that the degree of renal damage was markedly reduced in the pre-treated kidneys with PEG-AuNPs; these results are consistent with Chithrani and Chan (2007).

AuNPs were broadly used as an anti-hyperglycemic and anti-inflammatory agent (Edrees et al. 2017) and may be potential therapeutic agents for ischemic stroke (Zheng et al. 2019). After renal I/R in diabetic mice, the renal function was assessed by measuring serum BUN and creatinine; the results showed a massive increase in BUN and creatinine which indicates severe renal failure; these biochemical results are consistent with the histological changes. These results are compatible with the findings of Sadek et al. (2013) and Yang et al. (2019) who recorded significant elevation of serum levels of BUN and creatinine in rats that undergo renal I/R. Treatment with PEG-AuNPs 40, 150, and 400 µg/kg showed a significant decrease in BUN and creatinine measured. AuNPs have a curative effect on renal function and significantly improve the result of the biochemical markers measured of the kidney (Chithrani and Chan 2007). Although reperfusion is necessary for the survival of ischemic tissue, reoxygenation induces additional cellular injury. This reperfusion injury is due to the generation of ROS and oxidative stress. The level of MDA, end-product of lipid peroxidation, and biomarker of oxidative stress was significantly increased in the DI/R group (Rahmania et al. 2017) and was decreased in the 40, 150, and 400 µg/kg. A recent study reported that lipid peroxidation levels were elevated in STZ-induced diabetic rats (Jin et al. 2008). Moreover, the concentration of renal GSH, SOD, and GPx of the diabetic renal I/R group was significantly decreased, demonstrating the overproduction of ROS (Jin et al. 2008). The groups treated with PEG-AuNPs 40, 150, and 400 µg/kg showed a significant increase in the concentration of antioxidants (GSH, SOD, and GPx). According to these findings, we can conclude that AuNPs exhibited an antioxidant effect. This result is consistent with previous reports in showing that AuNPs have a potential antioxidant impact, by decreasing the levels of reactive oxygen and reducing oxidative damage without having toxic effects (Muller et al. 2017, Haupenthal et al. 2020). Moreover, PEG-AuNPs reduced lipid peroxidation and ROS generation that was accomplished in diabetic treated mice (BarathManiKanth et al. 2010); the anti-oxidative and anti-hyperglycemic effect of gold nanoparticles was demonstrated in STZ-induced diabetic rats.

Nrf2 is a crucial element of the systemic defense against oxidative stress and the related inflammatory responses (Li et al. 2018). Nrf2 activation reduces ROS production and redox damage (Kovac et al. 2015). Recent research explored that AMPK participates in both energy metabolism and redox balance by activating Nrf2 and revealed that Nrf2 is a downstream signal of AMPK (Mo et al. 2014). In the kidney, activating AMPK and Nrf2 leads to the prevention of renal lipid accumulation by reducing the fatty acid synthesis and increasing the concentration of GSH as well as activating SOD and GPx enzyme (Juszczak et al. 2020). In addition, upregulation of HO-1 by Nrf2 as in our results has prominent anti-inflammatory and antioxidant properties (Johnson et al. 2008). An earlier study established that the unstable redox state after I/RI can be returned by the Nrf2 and kidney injury can be weakened by improving ROS detoxification (Kaspar et al. 2009). The Nrf2 mechanism is vital in protecting the kidney against I/RI through activation of PI3K/AKT signaling pathways and diminished the inflammatory and oxidative damages (Nakaso et al. 2003). However, the data of current research recommended that intravenous injection of PEG-AuNPs 40, 150, and 400 µg/kg activated Nrf2 in an AMPK-dependent manner and suppressed oxidative stress and energy metabolism disruptions and consequently prevented renal damage. It was proved that AuNPs increased Nrf2 levels, which induces signaling of antioxidant genes including GPx and HO-1. This increase in Nrf2 may be due to the effect of AuNPs on thiol bonds of Kelch-like ECH-associated protein 1 (KEAP1) that captured Nrf2. This interaction becomes weakened and allows Nrf2 molecules to escape from KEAP1-mediated capture and accumulate in the cells exposed to oxidative stress. This process enhances cellular anti-oxidative defenses (Zhang and Hannink 2003). Also, the anti-inflammatory properties of AuNPs may be due to reduced free radical levels, since activated macrophages and neutrophils produce ROS which is the major cause of AKI. Podocytes and proximal tubular cells of the kidney are strongly express AMPK. DN-induced AMPK dysregulation was observed, while the activation of AMPK ameliorates the pathological features of diabetic renal ischemia. However, in the diabetic renal I/R injury, phosphorylation of AMPK was inhibited, which was consistent with studies by Viollet et al. (2009) and Guo et al. (2015). Also, the present study demonstrated that I/R was unable to activate the AMPK signaling pathway efficiently in diabetes. Our results confirmed that a high level of insulin and hyperglycemia decreases AMPK activity through phosphorylation of AMPK subunits by the PI3K/AKT pathway (Juszczak et al. 2020), where the protein levels of AMPK and PI3K/AKT were downregulated after 30 min of renal ischemia and 48 h reperfusion. The energy sensor AMPK regulates cell death and survival and can be stimulated by PEG-AuNPs and exert cellular defense.

Ischemic AKI involves activation and generation of cytokines including TNF-α, IL-1β, and infiltration of the kidney by macrophages (Bonventre and Yang 2011). In this study, it was also found that there was a significant increase in TNF-α and IL-1β levels in diabetic renal I/R. Previous studies have also stated that ROS increased nuclear factor-kappaB (NF-κB) production that stimulates the secretion of IL-1β, TNF-α, and other inflammation mediators (Liu et al. 2017). The gold nanoparticles are considered anti-inflammatory mediators due to their ability to diminish the expression of NF-κB and reduce subsequent inflammatory responses (Jeon et al. 2003). A previous study showed that PEG coating NPs does not induce a significant inflammatory response and regulate the secretion of TNF-α and IL-1β and indicated the role of AuNPs in reducing the levels of inflammation (Chen et al. 2017). Also, the anti-inflammatory properties of PEG-AuNPs arise from the ability of AuNPs to diminish the generation of ROS from macrophages and neutrophils (Haupenthal et al. 2020). Our results are in consistent with recent study where, IL-1β and TNF-α level in groups received PEG-AuNPs at concentrations of 40, 150 and 400 µg/kg were reduced, compared with non-treated I/R in diabetic mice. A preceding study showed that IL-1 β could aggregate around AuNPs and inhibit the biological activities of IL-1 β, and cell damage was ameliorated (Sumbayev et al. 2013). Moreover, a high level of HO-1 expression which is associated with Nrf2 activation causes inhibition in NF-κB signaling results in the reduced inflammatory response and these are in line with our finding (Ahmed et al. 2017).

## Conclusion

According to our results, it can be concluded that the pre-treatment of mice with PEG-AuNPs reduced severe histopathological changes of renal I/R including necrotic and apoptotic injury through the activation of phosphorylated AMPK that is critical to suppress mitochondrial dysfunction and excessive production of mitochondrial ROS after renal I/R. To the best of our knowledge, the present study was the first to investigate the effects of PEG-AuNPs on the activation of AMPK via the PI3K/AKT pathway in renal I/R in diabetic mice. PEG-AuNPs exhibited a valuable targeted therapeutic candidate in the treatment of renal I/R injury in diabetic mice–induced AKI.

## Data Availability

The datasets analyzed during the current study are available from the corresponding author on reasonable request.
